# Genome Sequence of *Sporolactobacillus laevolacticus* DSM442, an Efficient Polymer-Grade d-Lactate Producer from Agricultural Waste Cottonseed as a Nitrogen Source

**DOI:** 10.1128/genomeA.01100-13

**Published:** 2013-12-26

**Authors:** Hongyan Wang, Limin Wang, Jiansong Ju, Bo Yu, Yanhe Ma

**Affiliations:** CAS Key Laboratory of Microbial Physiological and Metabolic Engineering, Institute of Microbiology, Chinese Academy of Sciences, Beijing, Chinaa; Tianjin Institute of Industrial Biotechnology, Chinese Academy of Sciences, Tianjin, Chinab; College of Life Science, Hebei Normal University, Shijiazhuang, Chinac

## Abstract

*Sporolactobacillus laevolacticus* DSM442 is an efficient polymer-grade d-lactic acid producer from low-cost agricultural waste cottonseed powder as the sole nitrogen source. Here we present a 3.59-Mb assembly of its genome sequence, which might provide useful information to further improve the strain for higher production titers.

## GENOME ANNOUNCEMENT

The microorganism *Sporolactobacillus laevolacticus* was first isolated and described as *Bacillus laevolacticus* by Nakayama and Yanoshi in 1967 (1). Andersch et al. reclassified *Bacillus laevolacticus* as *Sporolactobacillus laevolacticus* according to phylogenetic analyses, as well as chemotaxonomic and physiological characterizations in 1994 (2). *S. laevolacticus* is a Gram-positive and motile microorganism and also shows microaerophilic and catalase-negative properties (3, 4). Previous studies of *S. laevolacticus* suggested that it is a homofermentative microorganism that completely converts glucose to d-lactic acid (3, 5). Recent study has shown that *S. laevolacticus* DSM442 can use cottonseed powder as the sole nitrogen source to produce d-lactic acid of high optical purity. A high concentration (144.4 g/liter) was obtained during fed-batch fermentation, with an average productivity of 4.13 g/(liter·h) and a high yield of 0.96 g/g glucose, indicating the predominant potential of *S. laevolacticus* DSM442 (6).

Here, we present the draft genome sequence of *S. laevolacticus* DSM442 obtained by using the Illumina HiSeq 2000 system (300-bp paired-end sequences), which was performed by the Chinese National Human Genome Center at Shanghai, China. With the Velvet program (7), we obtained 8,499,698 high-quality-read base pairs, which provided 474-fold coverage. Through data assembly, we obtained 61 contigs with a contig *N*_50_ of 112,365 bp. The average length of each contig is 58,855 bp and the largest one is 306,117 bp, with a total length of 3.59 Mb.

Gene prediction was performed in line with the predicted result of Glimmer 3.02, Genemark, and the Z-Curve program software, and 3,636 genes were predicted. The GC content of the predicted gene is 43.7%, and the average length of coding sequences (CDS) is 830 bp. The maximum CDS length is 7,092 and the minimum is 114 bp. There were 60 tRNA sequences found by tRNAscan and 9 rRNA sequences predicted via RNAmmer (8). Gene function annotations were made through searching the NCBI nucleotide and KEGG protein databases as well as the SEED protein databases of NCBI (9). The clusters of orthologous groups (COG) classification was performed using the CDD databases. The metabolic pathways of *S. laevolacticus* DSM442 were built through the KEGG databases. The annotation results showed that 2,688 proteins have clear biological functions, of which 1,737 proteins have KEGG orthologs and 2,683 proteins have COG classifications. All the matched homologous proteins are derived from 412 species, including *S. inulinus* CASD, which has the highest percentage, reaching 53.2%. *S. laevolacticus* DSM442 is predicted to possess complete metabolic pathways, including those for glycolysis, the tricarboxylic acid cycle, and the pentose phosphate pathway. Additionally, there are 107 genes annotated for inorganic ion transport and metabolism, including Na/Pi cotransporter II-like protein and calcium-transporting ATPase (EC 3.6.3.8), which might provide useful information to further increase the bacterium’s capacity to withstand salt duress and thus enhance lactate production.

### Nucleotide sequence accession numbers.

This whole genome shotgun project has been deposited at DDBJ/EMBL/GenBank under the accession number AWTC00000000. The version described in this paper is version AWTC01000000.
